# State-Specific Prevalence of Obesity Among Children Aged 2–4 Years Enrolled in the Special Supplemental Nutrition Program for Women, Infants, and Children — United States, 2010–2016

**DOI:** 10.15585/mmwr.mm6846a3

**Published:** 2019-11-22

**Authors:** Liping Pan, Heidi M. Blanck, Sohyun Park, Deborah A. Galuska, David S. Freedman, Anna Potter, Ruth Petersen

**Affiliations:** ^1^Division of Nutrition, Physical Activity, and Obesity, National Center for Chronic Disease Prevention and Health Promotion, CDC; ^2^Office of Policy Support, Food and Nutrition Service, U.S. Department of Agriculture, Alexandria, Virginia.

Obesity negatively affects children’s health because of its associations with cardiovascular disease risk factors, type 2 diabetes, asthma, fatty liver disease, victimization stemming from social stigma and bullying, and poor mental health (e.g., anxiety and depression) (*1*). Children who have overweight or obesity in early childhood are approximately four times as likely to have overweight or obesity in young adulthood as their normal weight peers (*2*). Obesity prevalence is especially high among children from low-income families (*3*). In 2010, the overall upward trend in obesity prevalence turned downward among children aged 2–4 years enrolled in the Special Supplemental Nutrition Program for Women, Infants, and Children (WIC), a program of the U.S. Department of Agriculture (USDA); prevalence decreased significantly in all racial/ethnic groups and in 34 of the 56 WIC state or territory agencies during 2010–2014 (*4*). A more recent study among young children enrolled in WIC reported that the overall obesity prevalence decreased from 15.9% in 2010 to 13.9% in 2016 and statistically significant decreases were observed in all age, sex, and racial/ethnic subgroups (*3*). However, this study did not provide obesity trends at the state level. In collaboration with USDA, CDC used data from the WIC Participant and Program Characteristics (WIC PC) to update state-specific trends through 2016. During 2010–2016, modest but statistically significant decreases in obesity prevalence among children aged 2–4 years enrolled in WIC occurred in 41 (73%) of 56 WIC state or territory agencies. Comprehensive approaches that create positive changes to promote healthy eating and physical activity for young children from all income levels,* strengthen nutrition education and breastfeeding support among young children enrolled in WIC, and encourage redemptions of healthy foods in WIC food packages could help maintain or accelerate these declining trends.

As a federal grant program, WIC is administered by states, territories, and Indian Tribal Organizations to provide supplemental nutritious foods, breastfeeding support, health care referrals, and nutrition education for low-income children aged <5 years and pregnant, postpartum, or breastfeeding women. WIC PC is a biennial census in even years of all participants certified to receive WIC benefits. WIC state and territory agencies extract WIC PC data in April of the reporting year. To be eligible for WIC, participants must live in the states in which they apply, have gross household income ≤185% of the federal poverty guidelines or be eligible for other programs (e.g., Supplemental Nutrition Assistance Program, Medicaid, and Temporary Assistance for Needy Families), and be at nutrition risk.^†^ Children’s weight and height are measured by WIC staff members during certification and recertification clinical visits.^§^

Obesity was defined as a body mass index ≥95th percentile for age and sex on the 2000 CDC growth charts.^¶^ To estimate relative change in obesity prevalence during 2010–2016, a log binomial regression analysis was performed for each WIC state or territory agency to obtain the prevalence ratio from 2010 to 2016 adjusted for age, sex, and race/ethnicity, using SAS software (version 9.4; SAS Institute). An obesity trend was considered statistically significant if the two-sided p-value was <0.05 in state-level log binomial regression model including all years of data. For absolute change in obesity prevalence, marginal effect was obtained from state-level logistic regression using the Margins package in R software (version 3.6; R Foundation for Statistical Computing) to show the adjusted prevalence difference from 2010 to 2016.

The final analytic sample included 12,403,629 children aged 2–4 years enrolled in the program from WIC agencies in 50 states, the District of Columbia, and five U.S. territories in 2010, 2012, 2014, and 2016. Among approximately 12.6 million original enrollees, a total of 171,272 (1.4%) children whose age, sex, weight, height, or body mass index were missing and 44,578 (0.4%) children whose anthropometric data were biologically implausible were excluded; biologically implausible z scores were defined as height for age <−5.0 or >4.0, weight for age <−5.0 or >8.0, and body mass index for age <−4.0 or >8.0.**

In 2010, crude obesity prevalence ranged from 9.6% (95% confidence interval [CI] = 9.3%–9.8%) in Colorado to 21.5% (95% CI = 21.2%–21.9%) in Virginia (Table). Obesity prevalence was ≥20% among children aged 2–4 years in three state or territory agencies (Alaska, Puerto Rico, and Virginia) and was <10% in only two WIC state agencies (Colorado and Hawaii). In 2016, crude obesity prevalences ranged from 7.8% (95% CI = 6.4%–9.2%) in the Northern Mariana Islands to 19.8% (95% CI = 18.8%–19.8%) in Alaska. Crude obesity prevalence among children aged 2–4 years was <20% in any state or territory and was <10% in six WIC state or territory agencies (Colorado, Guam, Hawaii, Northern Mariana Islands, Utah, and Wyoming).

**TABLE:** Prevalence of obesity among children aged 2–4 years enrolled in the Special Supplemental Nutrition Program for Women, Infants, and Children (WIC), by WIC state or territory agency — United States, 2010─2016

State	2010		2016		2016 versus 2010	
	No.	Crude prevalence % (95% CI)	No.	Crude prevalence % (95% CI)	Adjusted prevalence ratio*(95% CI)	Adjusted prevalence difference^†^ % (95% CI)
Alabama^§,¶^	45,743	15.8 (15.5 to 16.2)	42,671	16.3 (15.9 to 16.6)	1.03 (1.00 to 1.06)	0.5 (0.0 to 1.0)
Alaska**	10,108	21.2 (20.4 to 22.0)	5,983	19.8 (18.8 to 20.8)	0.92 (0.86 to 0.97)	–1.6 (–2.8 to –0.3)
Arizona**	72,933	15.0 (14.8 to 15.3)	58,054	12.1 (11.8 to 12.3)	0.81 (0.79 to 0.84)	–2.7 (–3.1 to –2.4)
Arkansas**	31,245	14.8 (14.4 to 15.2)	23,647	13.3 (12.8 to 13.7)	0.90 (0.87 to 0.94)	–1.4 (–2.0 to –0.8)
California**	583,008	18.4 (18.3 to 18.5)	495,095	15.5 (15.4 to 15.6)	0.86 (0.86 to 0.87)	–2.5 (–2.6 to –2.3)
Colorado**	39,612	9.6 (9.3 to 9.8)	31,307	8.1 (7.8 to 8.4)	0.85 (0.81 to 0.90)	–1.4 (–1.8 to –1.0)
Connecticut**	22,988	17.1 (16.6 to 17.6)	18,748	14.4 (13.9 to 14.9)	0.87 (0.83 to 0.91)	–2.2 (–2.9 to –1.5)
Delaware	7,650	18.4 (17.5 to 19.2)	6,906	16.2 (15.3 to 17.0)	0.93 (0.87 to 1.00)	–1.1 (–2.3 to 0.2)
District of Columbia**	5,182	14.4 (13.5 to 15.4)	5,181	11.4 (10.5 to 12.3)	0.83 (0.75 to 0.91)	–2.4 (–3.7 to –1.1)
Florida**	194,924	14.6 (14.4 to 14.7)	193,749	12.7 (12.6 to 12.9)	0.87 (0.86 to 0.89)	–1.8 (–2.0 to –1.6)
Georgia**	104,959	14.4 (14.2 to 14.6)	78,023	12.5 (12.3 to 12.8)	0.88 (0.86 to 0.90)	–1.8 (–2.1 to –1.4)
Hawaii	14,504	9.7 (9.3 to 10.2)	11,589	9.6 (9.1 to 10.1)	0.98 (0.91 to 1.06)	–0.2 (–0.9 to 0.6)
Idaho**	18,704	11.9 (11.5 to 12.4)	14,521	11.3 (10.8 to 11.8)	0.95 (0.89 to 1.00)	–0.6 (–1.3 to 0.1)
Illinois**	108,762	15.7 (15.5 to 15.9)	79,949	14.8 (14.6 to 15.0)	0.98 (0.96 to 1.00)	–0.3 (–0.6 to 0.1)
Indiana**	63,220	15.1 (14.8 to 15.4)	55,955	13.0 (12.7 to 13.2)	0.91 (0.88 to 0.93)	–1.4 (–1.8 to –1.0)
Iowa	29,481	15.6 (15.2 to 16.0)	24,427	15.2 (14.8 to 15.7)	1.00 (0.96 to 1.04)	0.0 (–0.6 to 0.6)
Kansas**	30,458	13.7 (13.4 to 14.1)	24,306	12.5 (12.1 to 12.9)	0.91 (0.87 to 0.95)	–1.3 (–1.8 to –0.7)
Kentucky**	45,761	18.2 (17.9 to 18.6)	38,361	15.9 (15.6 to 16.3)	0.88 (0.85 to 0.91)	–2.2 (–2.7 to –1.7)
Louisiana**	48,145	13.8 (13.5 to 14.1)	37,527	13.2 (12.9 to 13.6)	0.94 (0.91 to 0.98)	–0.8 (–1.2 to –0.3)
Maine**	10,410	15.2 (14.6 to 15.9)	8,233	13.9 (13.2 to 14.7)	0.92 (0.85 to 0.98)	–1.3 (–2.3 to –0.2)
Maryland**	51,280	17.1 (16.8 to 17.4)	50,469	15.6 (15.3 to 16.0)	0.92 (0.90 to 0.95)	–1.3 (–1.8 to –0.9)
Massachusetts**	49,178	17.1 (16.8 to 17.5)	41,740	16.4 (16.0 to 16.7)	0.94 (0.91 to 0.96)	–1.0 (–1.5 to –0.6)
Michigan**	85,293	14.4 (14.2 to 14.6)	84,387	13.3 (13.1 to 13.5)	0.95 (0.93 to 0.97)	–0.7 (–1.0 to –0.3)
Minnesota**	57,529	12.7 (12.4 to 13.0)	47,219	12.2 (11.9 to 12.5)	0.95 (0.92 to 0.98)	–0.6 (–1.0 to –0.2)
Mississippi**	36,519	14.9 (14.6 to 15.3)	28,493	14.4 (14.0 to 14.8)	0.96 (0.92 to 0.99)	–0.6 (–1.2 to –0.1)
Missouri**	50,575	14.4 (14.1 to 14.8)	43,404	12.3 (12.0 to 12.6)	0.86 (0.83 to 0.88)	–2.1 (–2.5 to –1.6)
Montana	7,194	13.4 (12.6 to 14.2)	6,647	12.1 (11.3 to 12.8)	0.89 (0.82 to 0.97)	–1.5 (–2.6 to –0.4)
Nebraska	15,622	14.4 (13.8 to 14.9)	13,807	15.2 (14.6 to 15.7)	1.05 (1.00 to 1.11)	0.8 (0.0 to 1.6)
Nevada**	25,855	15.0 (14.6 to 15.5)	24,493	11.6 (11.2 to 12.0)	0.80 (0.77 to 0.84)	–2.9 (–3.5 to –2.3)
New Hampshire	7,263	15.0 (14.1 to 15.8)	6,042	15.8 (14.9 to 16.7)	1.05 (0.97 to 1.14)	0.8 (–0.5 to 2.0)
New Jersey**	59,000	18.9 (18.6 to 19.2)	53,917	15.0 (14.7 to 15.3)	0.80 (0.78 to 0.82)	–3.9 (–4.3 to –3.4)
New Mexico**	21,968	15.7 (15.2 to 16.1)	18,619	12.1 (11.6 to 12.5)	0.77 (0.73 to 0.81)	–3.7 (–4.4 to –3.0)
New York**	186,760	16.1 (16.0 to 16.3)	182,401	13.7 (13.6 to 13.9)	0.88 (0.87 to 0.89)	–1.9 (–2.1 to –1.7)
North Carolina^§,¶^	89,798	13.9 (13.6 to 14.1)	97,286	14.2 (14.0 to 14.5)	1.04 (1.02 to 1.06)	0.6 (0.3 to 0.9)
North Dakota	5,484	14.5 (13.5 to 15.4)	4,723	14.3 (13.3 to 15.3)	0.99 (0.90 to 1.09)	–0.1 (–1.4 to 1.3)
Ohio	102,803	12.6 (12.4 to 12.8)	74,753	12.4 (12.2 to 12.6)	0.98 (0.96 to 1.01)	–0.2 (–0.5 to 0.1)
Oklahoma**	37,849	15.4 (15.1 to 15.8)	34,486	13.1 (12.8 to 13.5)	0.85 (0.82 to 0.88)	–2.4 (–2.9 to –1.8)
Oregon**	43,209	15.8 (15.5 to 16.2)	34,485	14.7 (14.4 to 15.1)	0.94 (0.91 to 0.97)	–1.0 (–1.5 to –0.5)
Pennsylvania**	96,762	12.8 (12.6 to 13.1)	80,202	12.2 (12.0 to 12.4)	0.96 (0.94 to 0.98)	–0.5 (–0.8 to –0.2)
Rhode Island**	10,783	16.4 (15.7 to 17.1)	6,984	15.4 (14.5 to 16.2)	0.93 (0.86 to 0.99)	–1.2 (–2.3 to –0.1)
South Carolina**	39,785	13.3 (13.0 to 13.7)	32,399	11.4 (11.1 to 11.8)	0.89 (0.85 to 0.92)	–1.5 (–2.0 to –1.0)
South Dakota	7,884	17.3 (16.5 to 18.1)	6,771	17.1 (16.2 to 18.0)	0.95 (0.88 to 1.02)	–0.8 (–2.1 to 0.4)
Tennessee**	57,153	16.0 (15.7 to 16.3)	51,157	14.6 (14.3 to 14.9)	0.92 (0.89 to 0.94)	–1.3 (–1.8 to –0.9)
Texas**	361,823	16.9 (16.8 to 17.0)	268,787	14.6 (14.4 to 14.7)	0.89 (0.88 to 0.90)	–1.9 (–2.0 to –1.7)
Utah**	26,045	12.5 (12.1 to 12.9)	21,599	7.9 (7.6 to 8.3)	0.64 (0.60 to 0.67)	–4.6 (–5.1 to –4.0)
Vermont	6,964	13.8 (13.0 to 14.7)	5,254	14.5 (13.5 to 15.4)	1.04 (0.95 to 1.13)	0.6 (–0.7 to 1.8)
Virginia^¶,^**	48,920	21.5 (21.2 to 21.9)	47,376	15.3 (14.9 to 15.6)	0.73 (0.71 to 0.75)	–5.8 (–6.3 to –5.3)
Washington**	78,336	14.9 (14.6 to 15.1)	69,870	13.3 (13.0 to 13.5)	0.89 (0.87 to 0.91)	–1.6 (–2.0 to –1.3)
West Virginia^§,¶^	17,669	14.4 (13.9 to 14.9)	14,222	16.6 (16.0 to 17.2)	1.15 (1.09 to 1.21)	2.2 (1.4 to 3.0)
Wisconsin**	48,511	15.2 (14.9 to 15.5)	37,116	14.3 (14.0 to 14.7)	0.94 (0.91 to 0.97)	–0.9 (–1.4 to –0.4)
Wyoming**	4,413	11.8 (10.9 to 12.8)	3,458	9.1 (8.1 to 10.1)	0.76 (0.67 to 0.87)	–2.8 (–4.2 to –1.5)
**Territory**						
American Samoa	3,221	14.6 (13.4 to 15.8)	2,824	13.7 (12.4 to 15.0)	0.94 (0.83 to 1.06)	–0.9 (–2.7 to 0.9)
Guam**	3,248	11.4 (10.3 to 12.5)	2,710	8.3 (7.3 to 9.4)	0.73 (0.62 to 0.85)	–3.1 (–4.6 to –1.6)
Northern Mariana Islands**	2,157	14.1 (12.6 to 15.6)	1,418	7.8 (6.4 to 9.2)	0.55 (0.45 to 0.68)	–6.4 (–8.4 to –4.4)
Puerto Rico**	70,699	20.3 (20.0 to 20.6)	63,251	12.0 (11.8 to 12.3)	0.60 (0.58 to 0.61)	–8.2 (–8.6 to –7.8)
U.S. Virgin Islands	2,093	12.4 (11.0 to 13.8)	1,593	13.1 (11.5 to 14.8)	1.07 (0.90 to 1.26)	0.8 (–1.4 to 3.0)

**Abbreviation:** CI = confidence interval.

* Obtained from log binomial regression model adjusted for age in month, sex, and race/ethnicity.

^†^ Calculated as 100 times the average marginal effect of year (2010 versus 2016) from logistic regression controlling for age, sex, and race. A negative value indicates that the prevalence decreased.

^§^ Statistically significant increase in obesity prevalence during 2010–2016 determined by trend test using log binomial regression model adjusted for age, sex, and race/ethnicity with all years of data included.

^¶^ Change in the data reporting system in 2016 might affect obesity prevalence.

** Statistically significant decrease in obesity prevalence during 2010–2016 determined by trend test using log binomial regression model adjusted for age, sex, and race/ethnicity with all years of data included.

During 2010–2016, statistically significant decreases in obesity prevalence occurred in 41 of 56 WIC state or territory agencies (p<0.05 for trend test) across all years (Table) (Figure). Adjusted obesity prevalences decreased by >3 percentage points in seven WIC state or territory agencies (Guam, New Jersey, New Mexico, Northern Mariana Islands, Puerto Rico, Utah, and Virginia); the largest significant decrease was in Puerto Rico, where adjusted obesity prevalence among WIC beneficiaries aged 2–4 years decreased by 8.2 percentage points from 2010 to 2016. Only three WIC state agencies reported significant increases in obesity prevalence across all years; adjusted obesity prevalence increased by 0.5 percentage points in Alabama, 0.6 percentage points in North Carolina, and 2.2 percentage points in West Virginia (Table).

**FIGURE:**
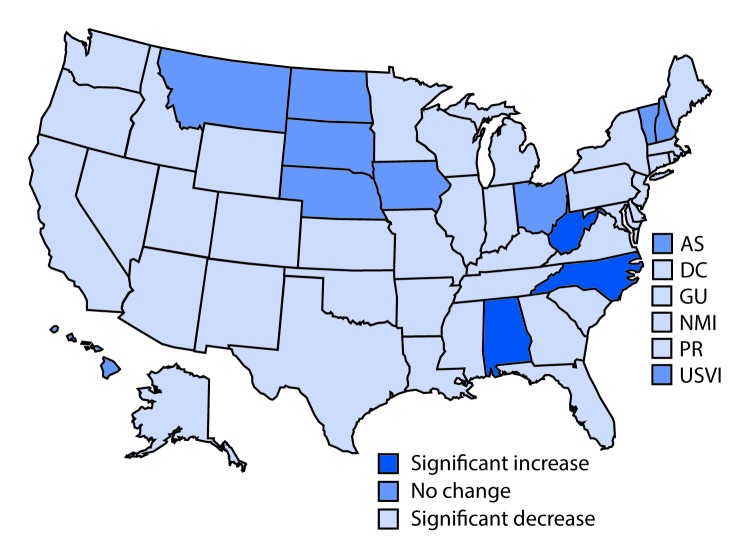
Changes* in obesity prevalence among children aged 2–4 years enrolled in the Special Supplemental Nutrition Program for Women, Infants, and Children (WIC), by WIC state or territory agency — United States, 2010─2016 **Abbreviations:** AS = American Samoa; DC = District of Columbia; GU = Guam; NMI = Northern Mariana Islands; PR = Puerto Rico; USVI = U.S. Virgin Islands. * Statistically significant changes were determined by trend tests using log binomial regression models adjusted for age, sex, and race/ethnicity with all years of data included.

## Discussion

These findings indicate statistically significant decreases in obesity prevalence during 2010–2016 among children aged 2–4 years enrolled in WIC in 41 (73%) of 56 WIC state or territory agencies. A previous study using these data reported that children aged 2–4 years in 34 (61%) of 56 WIC state or territory agencies experienced decreases in obesity prevalence during 2010–2014 (*4*). The present study found that obesity prevalence among children in this age group continued to decrease through 2016 in 33 of the 34 WIC state or territory agencies, with previous significant decreases and identified decreases during 2010–2016 in eight additional WIC agencies having no significant changes during 2010–2014. Although decreases in obesity prevalence in the present study were small, the trends in obesity prevalence among young WIC beneficiaries overall (*3*) and in the majority of states and territories were in contrast to the national trend, which was that obesity prevalence decreased for children aged 2–5 years from all income levels from 10.1% in 2007–2008 to 8.4% in 2011–2012 and then increased to 13.9% in 2015–2016 (*5*). Thus, even these small decreases indicate progress for this vulnerable WIC population.

The WIC program reaches low-income infants and children during the critical period of child growth. One factor that might have contributed to the observed decreases in obesity prevalence in WIC enrollees is the 2009 revisions to the WIC food packages (*6*), which was carried out to better align with nutrition research, the 2005 Dietary Guidelines for Americans (*7*), and the infant food and feeding practice guidelines of the American Academy of Pediatrics.^††^ The revised food packages include a broader range of healthy food options; promote fruit, vegetable, and whole wheat product purchases; support breastfeeding; and give WIC state and territory agencies more flexibility to accommodate cultural food preferences (*6*). The WIC package revisions had plausible impact on improving diet quality measured by the Healthy Eating Index–2010 scores among WIC children aged 2–4 years (*8*). In addition, the availability of healthier foods and beverages in authorized WIC stores has increased. Children enrolled in WIC consumed more fruits, vegetables, and whole grain products and less juice, white bread, and whole milk after the revisions (*9*) than they did before.

Additional contributors to these decreases in obesity prevalence might include other local, state, and national efforts and programs that affect changes in systems outside of WIC to improve diet quality and physical activity for young children from all income levels, including children enrolled in WIC. For example, CDC distributes funding on a competitive basis to state and local grantees to enable implementation of childhood obesity prevention strategies through increasing involvement of health care providers, community leaders, and early care and education providers (*10*). Many of the funding recipients focus on both population-level strategies such as state-level standards that can potentially benefit all children and more directed interventions for populations at the highest risk (*10*). In addition, CDC provides technical support for states to promote maternity care policies and practices to support breastfeeding in birthing facilities and workplaces.^§§^ CDC also provides support for states and communities to implement nutrition, breastfeeding support, physical activity, and screen time standards in early care and education systems and setting.^¶¶^

The findings in this report are subject to at least four limitations. First, approximately 15% fewer children were enrolled in WIC in 2016, compared with 2010 (*3*), and characteristics of those enrolled in WIC might have changed over time. Although the trend analyses adjusted for age, sex, and race/ethnicity, other unmeasured factors might have contributed to the declining trends in obesity. Second, the findings might not apply to all low-income children because children enrolled in WIC might be systematically different from others who are eligible but not enrolled. Third, the study findings cannot be applied to U.S. children from families with other income levels. Finally, certain states changed their data reporting systems in recent years, which might have affected obesity trends. Strengths of this study include the use of a large sample of children enrolled in WIC as derived from census data, allowing for stratification by state or territory and the use of measured weight and height data.

Despite these recent decreases in obesity among children enrolled in WIC, obesity prevalence remained high in most states in 2016. Multiple approaches are needed to address and eliminate childhood obesity. The National Academy of Medicine and other groups have recommended a comprehensive and integrated approach that calls for positive changes in physical activity and food and beverage environments in multiple settings including home, early care and education (e.g., nutrition standards for food served), and community (e.g., neighborhood designs that encourage walking and biking) to promote healthy eating and physical activity for young children. Further implementation of these positive changes across the United States could further the decreases in childhood obesity.

**Summary**

**What is already known about this topic?**

Among children aged 2–4 years enrolled in the Special Supplemental Nutrition Program for Women, Infants, and Children (WIC), obesity prevalence decreased from 15.9% in 2010 to 13.9% in 2016 and during 2010–2014, decreased in 34 of the 56 WIC state or territory agencies.

**What is added by this report?**

During 2010–2016, statistically significant decreases in obesity prevalence among WIC beneficiaries aged 2–4 years occurred in 41 of 56 WIC state or territory agencies; obesity prevalence ranged from 7.8% to 19.8%.

**What are the implications for public health practice?**

To accelerate these trends, expanded positive changes in multiple settings to promote healthy eating and physical activity for young children are needed.

## Footnotes

* https://www.nap.edu/read/13275/chapter/1.

† https://www.fns.usda.gov/wic/wic-eligibility-requirements.

§ https://wicworks.fns.usda.gov/wicworks/Sharing_Center/PA/Anthro/lib/pdf/Anthropometric_Training_Manual.pdf.

¶ https://www.cdc.gov/growthcharts/cdc_charts.htm.

** https://www.cdc.gov/nccdphp/dnpao/growthcharts/resources/sas.htm.

†† https://www.aap.org/en-us/advocacy-and-policy/aap-health-initiatives/HALF-Implementation-Guide/Age-Specific-Content/Pages/Infant-Food-and-Feeding.aspx/.

§§ https://www.cdc.gov/nccdphp/dnpao/state-local-programs/breastfeeding.html.

¶¶ https://www.cdc.gov/obesity/strategies/childcareece.html.
